# An Artificial Placenta Experimental System in Sheep: Critical Issues for Successful Transition and Survival up to One Week

**DOI:** 10.3390/biomedicines11030702

**Published:** 2023-02-24

**Authors:** Elisenda Eixarch, Miriam Illa, Raquel Fucho, Kambiz Rezaei, Ameth Hawkins-Villarreal, Sara Bobillo-Pérez, Paula C. Randanne, Miguel Moran, Marina Chorda, Sergio Sanchez-Martinez, Yolanda J. D. de Roo-Puente, Maria del Mar Velilla, Ruth del Rio, Marc Gallego, Daniel Sanin-Ramirez, Victor Narvaez, Fatima Crispi, Elisenda Bonet-Carne, Eduard Gratacos

**Affiliations:** 1 BCNatal Fetal Medicine Research Center, Hospital Clínic and Hospital Sant Joan de Déu, Universitat de Barcelona, 08028 Barcelona, Spain; 2 Institut d’Investigacions Biomèdiques August Pi i Sunyer (IDIBAPS) and Centre for Biomedical Research on Rare Diseases (CIBERER), 08036 Barcelona, Spain; 3 Institut de Recerca Sant Joan de Déu, Esplugues de Llobregat, 08950 Barcelona, Spain; 4 Cardiovascular Surgery Unit, Hospital Universitario Virgen del Rocio, 41013 Sevilla, Spain; 5 Fetal Medicine Service, Obstetrics Department, Hospital “Santo Tomás”, University of Panama, Panama City 07127, Panama; 6 Paediatric Intensive Care Unit, Hospital Sant Joan de Déu, University of Barcelona, Esplugues de Llobregat, 08950 Barcelona, Spain; 7 Pediatric Cardiology Department, Hospital Sant Joan de Déu, University of Barcelona, Esplugues de Llobregat, 08950 Barcelona, Spain; 8 Perfusion Department, Hospital Clinic, University of Barcelona, Esplugues de Llobregat, 08036 Barcelona, Spain; 9 Translational Computing in Cardiology, Institut d’Investigacions Biomèdiques August Pi i Sunyer (IDIBAPS), Esplugues de Llobregat, 08036 Barcelona, Spain; 10 Neonatal Unit, Hospital Sant Joan de Déu, University of Barcelona, Esplugues de Llobregat, 08950 Barcelona, Spain; 11 Barcelona Tech, Universitat Politècnica de Catalunya, Esplugues de Llobregat, 08034 Barcelona, Spain

**Keywords:** artificial placenta, artificial womb, fetal lamb

## Abstract

Objective: To describe the development of an artificial placenta (AP) system in sheep with learning curve and main bottlenecks to allow survival up to one week. Methods: A total of 28 fetal sheep were transferred to an AP system at 110–115 days of gestation. The survival goal in the AP system was increased progressively in three consecutive study groups: 1–3 h (n = 8), 4–24 h (n = 10) and 48–168 h (n = 10). Duration of cannulation procedure, technical complications, pH, lactate, extracorporeal circulation (EC) circuit flows, fetal heart rate, and outcomes across experiments were compared. Results: There was a progressive reduction in cannulation complications (75%, 50% and 0%, *p* = 0.004), improvement in initial pH (7.20 ± 0.06, 7.31 ± 0.04 and 7.33 ± 0.02, *p* = 0.161), and increment in the rate of experiments reaching survival goal (25%, 70% and 80%, *p* = 0.045). In the first two groups, cannulation accidents, air bubbles in the extracorporeal circuit, and thrombotic complications were the most common cause of AP system failure. Conclusions: Achieving a reproducible experimental setting for an AP system is extremely challenging, time- and effort-consuming, and requires a highly multidisciplinary team. As a result of the learning curve, we achieved reproducible transition and survival up to 7 days. Extended survival requires improving instrumentation with custom-designed devices.

## 1. Introduction

In the developed world, extreme prematurity is the leading cause of neonatal mortality and morbidity [1,2]. Among extreme preterm neonates, those born near viability have low survival rates, ranging from 28% at 23 weeks to 83% at 26 weeks [3] and a high rate of major sequelae, from 89% at 23 weeks to 71% at 26 weeks [4], including chronic lung and cardiovascular disease and motor and cognitive deficits [4]. These figures have only slightly improved over the recent decades [3,5]. One of the multiple reasons for the poor outcomes is the immature fetal pulmonary system, which represents a biological barrier for ventilation-based life support, as currently used in neonatal units. An artificial placenta (AP) aims at maintaining a preterm newborn as a fetus, and not as a neonate, in an intrauterine-like environment. Thus, this might avoid some of the complications of intensive neonatal support, potentially improve survival rates, and reduce major sequelae. At extremely low gestational ages, every extra day gained in the intrauterine life has enormous benefits in survival and morbidity [6]. Indeed, extending life under fetal conditions for a few weeks could allow substantial gains in maturation and postpone delivery to neonatal life at later gestational ages when mortality and morbidity rates are lower.

The concept of an AP is 60 years old [7]. After initial advances in the 1960s and 1970s, research was partially abandoned in the 1980s with the improvements in neonatal care. However, it was revived later, focusing on extremely or near-viable preterm newborns. In recent years, several groups [8,9] have provided evidence of the ability to maintain a fetus with AP support in experimental conditions. Flake et al. in Philadelphia [8] reported up to four weeks survival in an AP system. The pioneering work of these groups has paved the way for the first clinical trials in the coming years and has raised the need for further experimental research to refine knowledge on the impact on fetal and neonatal development. The development of an experimental AP system represents a great research challenge. Nonetheless, there is scarcely any research published providing information on the details and main difficulties found on setting up such a system.

In this study, we describe the setup of an AP system in sheep, the learning curve, and the main challenges encountered to achieve reproducible connection and survival in an AP system for up to one week.

## 2. Materials and Methods

A total of 28 fetal lambs from 28 pregnant ewes (Ripollesa sheep bred) were transferred to an AP system at 110–115 gestational days. Survival goal was increased progressively to 1–3 h, 4–24 h, and 48–168 h. Main variables affecting successful transition and survival were rigorously recorded: umbilical cord cannulation technique, cannulation procedure duration, technical complications, pH post-cannulation, circuit blood flow and fetal heart rate.

Time-dated pregnant sheep were used at gestational ages of 110–115 days (term 145 days), which corresponds to the mid to late canalicular phase of lung development [10]. Animal handling and all procedures were performed in accordance with applicable regulations and fulfilling ARRIVE guidelines [11] and with the approval of the Animal Experimental Ethics Committee of the University of Barcelona (67/20 P1 and 67/20 P2; 25 January 2021).

### 2.1. Surgical Procedure

Pregnant ewes were anesthetized with intramuscular administration of acepromazine (0.03 mg/kg), ketamine (5 mg/kg), midazolam (0.25 mg/kg) and buprenorphine (0.01 mg/kg) with maintenance of general anesthesia with isoflurane (2–3% in oxygen/air at 50% at 1 L/min) and propofol (2–4 mg/kg). Once jugular access was achieved, intravenous fluid reposition was administered (Ringer Lactate 1000 mL in 30 min) before starting the procedure to prevent hypotension. We collected 250 mL of blood from jugular vein using an aseptic technique that was immediately heparinized (5 UI/mL) and used for priming the AP circuit. Intraoperative hemodynamic monitoring included heart rate, blood pressure, and O_2_ saturation. Fetal viability was assessed by ultrasound and the estimated fetal weight was calculated [12]. A lower midline laparotomy was done to expose the uterus. Fetal ultrasound was performed through the uterine wall to evaluate fetal heart rate, sex, and position. Then, a small hysterotomy was performed to expose the umbilical cord. Before starting fetal manipulation, intramuscular fetal anesthesia (fentanyl 3 mcg/kg, midazolam 0.2 mg/kg and rocuronium 1 mg/kg) was administered.

### 2.2. Artificial Placenta System Components and Procedures

**Figure 1:**
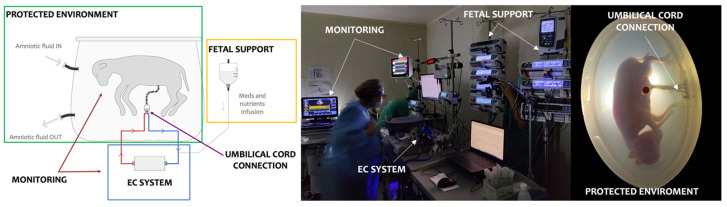
Main components of the artificial placental system. EC: extracorporeal circuit.

The main components of the AP experimental system are presented in Figure 1**.**

#### 2.2.1. Umbilical Cord Cannulation and Connection

Cannulae were placed in one vein and two arteries (Bio-Medicus^TM^ arterial cannulae 10 to 14 Fr depending on vessel size, Medtronic, Minneapolis, Minnesota, USA) using the bigger diameter fitting to each vessel. The umbilical cord cannulation technique was refined along experimental procedures. We started with a direct cut-down technique occluding the umbilical cord and posterior section, and after this procedure, the cannulation of the three vessels (2 arteries and 1 vein) was done. After several experiments, we moved to a sequential technique in which the umbilical cord connective tissue was dissected to expose umbilical vessels and 2/0 silk ligature was placed to individualize each vessel. Cannulae were inserted first in one vein and in one artery and then connected to the AP circuit. Once the connection was established, the second artery was cannulated and connected. We further refined this technique adding intensive vasospasm prevention. After cannulation and stabilizing sutures, an external fixation system was employed for all three cannulation techniques. In long-term experiments, the urachus in male fetuses was cannulated with 4–7 Fr catheter before umbilical cord vessel cannulation to avoid urine retention due to immaturity of male urethra [13]. After connection of the cannulae to the AP system, the fetus was transferred to the reservoir.

#### 2.2.2. Extracorporeal Circulation (EC) System

The EC circuit was pumpless and consisted of a low-resistance hollow fiber oxygenator, Quadrox-i Neonatal (Maquet, Rastatt, Germany) for short-term experiments (hours) and Quadrox-iD Pediatric (Maquet, Rastatt, Germany) for long-term experiments (days) connected to 1/4”ID × 1/16” PVC tubing (Sorin Group) for short-term experiments (hours) and 1/4”ID × 1/16” Bioline-coated tubing (Maquet, Rastatt, Germany) for long-term experiments (days), respectively. This was an arterial-venous EC circuit, with the two umbilical arteries merging in a single inflow tube towards the oxygenator, whose outflow port was connected to the umbilical vein. The priming volume of the entire circuit, including the oxygenator, was 90 mL (90–92) for short-term experiments and 127 mL (125–128) for long-term experiments. Before connection, maternal blood was heated through circulation in the oxygenator at 36 °C. Sweep gas supplied to the oxygenator was a mixture of medical air, oxygen, and nitrogen to achieve fetal blood gases of PaO_2_ 15–25 mmHg and PaCO_2_ 35–55 mmHg.

#### 2.2.3. Protected Environment

In the first stages of the study, a 20-L semiclosed heated reservoir filled with saline solution held at a constant of 38–40 °C was used. For mainly the long-duration experiments, the reservoir held a capacity of 10 L of synthetic amniotic fluid, and occasionally saline solution. The amniotic fluid was supplemented with ceftazidime 0.1 g/L and was continuously filtered and passed through UV irradiation to prevent bacterial colonization.

#### 2.2.4. Monitoring System

Post-membrane EC circuit flow was continuously measured using a clamp-on US-based flow sensor connected to a flowmeter module (ME6PXL sensor and TS410 flowmeter Transonic Systems Inc., Ithaca, NY, USA, respectively). Pre- and post-membrane pressure were monitored with MLT844 physiological pressure transducers from ADinstruments and MLT0670 Disposable BP Transducer from ADinstruments Inc, for short- and long-term experiments respectively. Fluid temperature in the amnio-bath was continuously monitored with an MLT1407 rectal thermocouple probe for large animals (ADinstruments Inc., Sydney, Australia). Heart rate, EC circuit blood flow rates, transmembrane pressure differential, sweep gas flow (GB100+, MCQ Instruments, Rome, Italy) and fetus environment fluid temperature were integrated and continuously recorded at a sampling rate of 100 Hz, and displayed in real time using LabChart 8 for research proposals. For the later experiments, the Quantum 12” Workstation Elite (Spectrum Medical, Gloucester, United Kingdom) was used for all sensor technology, which was directly connected to our in-house dashboard monitoring system.

#### 2.2.5. Fetal Support

Right after connection of the umbilical cord to the EC circuit, 15 mL/kg of packed red blood cells, hydrocortisone (3 mg) and meropenem (15 mg/kg) were administered. Hydrocortisone and meropenem were maintained during 3 days at 2 mg/kg/6 h and 15 mg/kg/8 h, respectively. A heparin bolus of 100 IU/kg was also administered at the time of connection followed by continuous infusion of 20 IU/kg/h that was adjusted to obtain an activated clotting time (ACT) levels of 200–250 s measured by Hemochron Signature Elite (Accriva Diagnostics, San Diego, CA, USA). Prostaglandin E1 perfusion was started at 0.1 mcg/kg/min and progressively diminished to the minimum doses required to maintain ductus arteriosus permeability assessed by echocardiography every 12 h.

Glucose 20% infusion was administered at 50 mL/kg/day and at 6 h after AP system connection total parenteral nutrition was started as follows: glucose 10 g/kg/d (7 mg/kg/min); proteins 3 g/kg/day; lipids 0.3–0.4 g/kg/day; Na 4 mEq/kg/day; K 1.5 mEq/kg/day; Ca 3.5 mEq/kg/day; P 1.75 mEq/kg/day; Mg 0.5 mEq/kg/day, and oligoelements and vitamins to obtain a total caloric goal of 48 kcal/kg/day. Daily adjustments were made to maintain ion levels in fetal blood.

Packed maternal red blood cells was transfused as required (10 mL/kg) to maintain fetal hemoglobin levels above 9 g/dL. In long-term experiments, erythropoietin (400 IU/kg) was administered intravenously daily from the first day of the experiment.

Pre- and post-membrane blood samples were analyzed every 1–2 h for blood gas, electrolyte and coagulation values using EPOC^®^ system.

Fetal sedation with midazolam (0.2 mg/kg/h), fentanyl (2 mcg/kg/h) or cisatracurium (4 mcg/kg/h) was used as required. Fetuses were maintained in the AP system until the survival goal and subsequently euthanatized with pentobarbital (200 mg/kg intravenously).

#### 2.2.6. Statistical Analyses

The statistics presented in the tables were calculated using jamovi [14]. Categorical variables are expressed as counts and percentages, and group differences are assessed using the χ2 test. Continuous variables are reported as means ± SEM and group differences were assessed using Student’s *t*-test, ANOVA (when normality distribution was confirmed by Kolmogorov–Smirnov test) or Kruskal–Wallis test. A *p* value of < 0.05 was considered statistically significant.

## 3. Results

Gestational age at AP connection was 112 ± 0.6 days with an ultrasound-estimated fetal weight of 1681 ± 76.9 g and a proportion of male/female of 13/15. The number of animals that achieved the survival goal for each group increased from 25% in the 1–3 h group to 70% in 4–24 h group and 80% in 48–168 h group (*p* = 0.045).

Table 1 summarizes experimental outcomes in the three groups. The first group of cases (1–3 h group) was associated with a high rate of cannulation accidents, air bubbles in the EC circuit and, more rarely, thrombotic complications, of which a significant number of cases died shortly after connection. The rate of complications was substantially lower in the subsequent 3–24 h and 48–168 h survival groups. In the latter group, all animals could be cannulated without complications. No differences were found in the overall surgical time or cannulation time, nor in fetal heart rate before connection.

**Figure 2:**
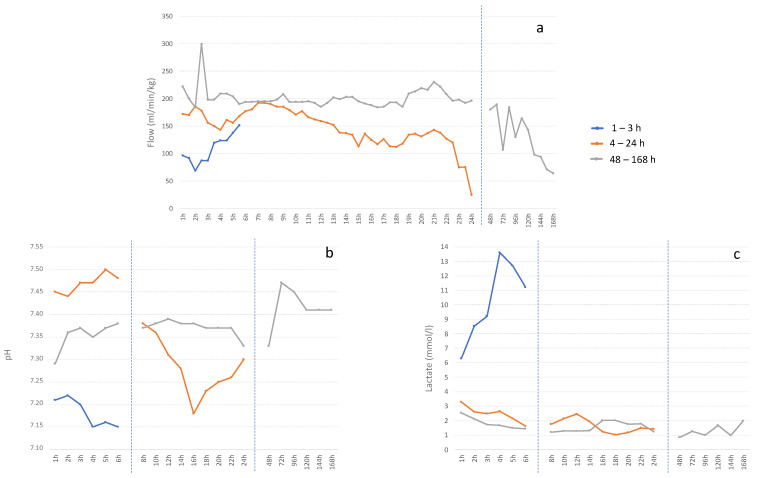
Representative data of the longitudinal evolution in one animal of each experimental group. Graphics showing mean value for each time point of (**a**) circuit flow normalized by fetal weight (ml/kg/min), (**b**) pH and (**c**) lactate (mmol/L).

**Figure 3:**
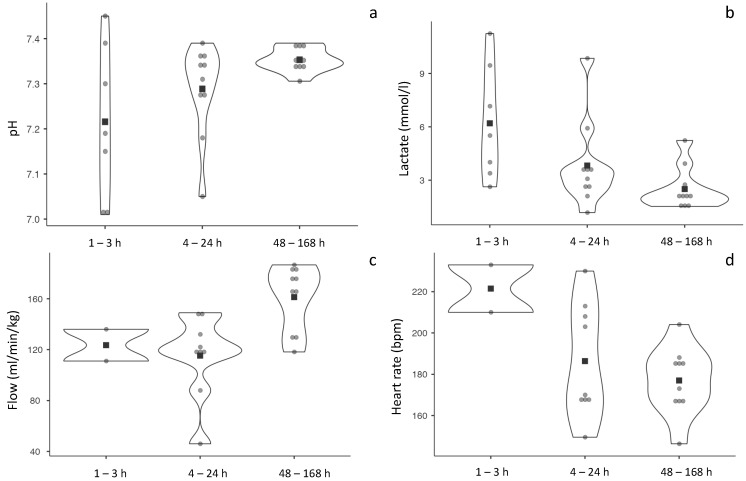
Distribution of pH, lactate, circuit flow and heart rate during the whole experiment in the experimental groups. Violin plots showing (**a**) pH, (**b**) lactate (mmol/L), (**c**) circuit flow normalized by fetal weight (mL/kg/min), and (**d**) fetal heart rate (BPM). Each point (•) represents mean value of each animal considering whole experiment and black square (■) mean value of each experimental group.

Experiments showed a trend for a progressive improvement of pH post-cannulation (*p* = 0.161) and a significant improvement in EC circuit flow after 30 min of connection (*p* = 0.003) across the study groups. Figure 2 shows data of the longitudinal evolution of these parameters in one fetal lamb of each experimental group. As shown in Figure 3, experiments with 48–168 h survival have a more homogenous distribution of pH, lactate, circuit flow and heart rate. Among the 48–168 h survival group, 4 out of 10 fetal lambs presented complications after the first day, consisting in cannulation accidents leading to interruption of flow (n = 2) and sepsis (n = 2). In three lamb fetuses in the 48–168 h group, fetal hydrops was observed at the end of the experiment.

## 4. Discussion

In this paper, we have described the successful development of an experimental prototype of AP in fetal lamb that achieved a reproducible transition from uterus to AP and survival in the system for up to 7 days.

The design of our experimental setup was inspired by previous reports published by other groups. The Children’s Hospital of Philadelphia (CHOP) developed the EXTEND system [15] with underwater maintenance and umbilical cord cannulation [16,17], pumpless extracorporeal circulation and very low priming volume oxygenating membrane (38 mL) to mimic feto-placental conditions, achieving 3–4 week survival in 95- to 110-day lambs [18], with no major effects on brain [19] or cardiovascular function—some reductions in contractility [18]—near normal growth, lung histology and function [8] and gastrointestinal tract [20]. Tohoku University (Japan) and Western Australia University have a system similar to CHOP in 95-day lambs [21,22], reporting up to 5 days survival with normal growth and cardiac function, no lung damage, but some hypoxic–ischemic white matter lesion (14.3%). As in the other models, some cases develop an inflammatory status, generalized or limited to the lungs [23]. The University of Michigan used a different approach: venous–venous cannulation of the jugular and umbilical veins in 110–130 days lambs [24,25] with a rotatory pump and the fetus intubated with fluid-filled lungs [26]. They reported up to 7 days of survival [25] and improved lung development compared to newborns with mechanical ventilation [26,27]. Cerebral oxygenation was maintained [28] with no major effects in brain histology, folding or white matter [29], normal spleen [30] and gastrointestinal system [31]. This study complements previous studies and contributes on this topic by providing practical information that may be useful for other researchers.

Cannulation and the EC system are possibly the main limitations in the setup of an AP experimental system. Commercial cannulae and EC circuits are too large and not designed for working with an umbilical cord, and this entails several challenges. First, to achieve a reproducible technique for umbilical cord cannulation, an intensive training of the surgical team with ex vivo models was required. Despite this training, 15 in vivo experiments were required to achieve reproducible results. Cannulation entails a combination of skills and delicate manipulation of the umbilical vessels, which are physiologically prone to vasospasm. Cannulation of the urachus to avoid urine retention of male fetuses is another requirement in this model, due to the immaturity of male urethra lamb during fetal period [13]. However, this might not represent a problem in the human fetus. Secondly, transfer to and maintenance in the protected environment (bag/box) is difficult because of the size of the cannulae. While accidents during transportation are minimized and eventually disappear with the learning curve, cannula size remains a problem when survival time is extended and leads to potentially fatal umbilical cord accidents, as reported by previous groups [16] and observed in this study. Thirdly, there is a need for continuous anticoagulation. In our experience, anticoagulation times (ACT) of 150–250 s allowed the maintaining of the functional oxygenator membrane without hemorrhagic complications in most cases. However, achieving optimal ranges can be challenging due to variable need for heparin in each individual case. A further limitation is that the fetal sheep above 85 days of pregnancy does not represent a good model for intracranial hemorrhage [32]. Better EC circuit and oxygenator membrane systems that require less anticoagulation are a critical need when considering future clinical applications.

In this study, we used a semiclosed system with continuous flow circulation for the protected fetus environment, which required continuous antibiotic treatment to avoid infections. However, a closed fetal container is a more desirable solution. Flake et al. reported the absence of infections with the use of such systems [8]. After successful cannulation and transfer, fetal medical support and monitoring of the fetus transferred to the AP system was performed with a strict protocol inspired in neonatal intensive care unit (ICU) management. Fetal nutrition and metabolic adjustments can be performed as per neonatal protocols, and after an initial adaptation and in the absence of complications, the fetus displayed a remarkable hemodynamic and metabolic stability. A further challenge for achieving long-term survival in the AP system is to reproduce a near-physiological circulation, i.e., resembling feto-placental pressure and volume conditions. This seems to be essential to avoid congestive cardiac failure and hydrops, as has been observed in previous studies [17] and in ours. This problem can be overcome with several adaptations in the oxygenator membrane and EC circuit, but this discussion lies beyond the scope of this report.

From a clinical standpoint, AP systems could revolutionize the current management of extremely preterm infants. The challenge of applying an AP in clinical practice is formidable. It is likely that the first clinical trials will face unexpected new challenges. In the beginning, an AP would possibly be limited to newborns in the limit of viability associated with very high rates of mortality and morbidity. However, once the system proves its feasibility and safety, its use could be extended to later gestational ages or new approaches for fetal surgery. From a research and innovation point of view, an AP generates a new scientific interdisciplinary field, where current notions of fetal medicine merge with intensive neonatal care. This opens a new vision for clinicians and researchers on fetal and neonatal medicine, but it also involves social sciences, data science and medical technology. An AP might complement other advances in early human development research, including organ-on-a-chip research, which could contribute with relevant information on placental functions (i.e., hormonal, immunological, nutritional) and further refinement of some components of the AP system. Developing solid experimental platforms for AP will be essential for the advancement of research in this field and will open new opportunities for fetal development and therapy, from basic to preclinical research. Increasing survival up to 28 days and minimizing adverse effects in fetal development would be essential to support the preliminary design of clinical trials.

The main limitations of this study are that most fetuses were 110–115 days’ gestational age. The mean fetal size at this gestational age is over 1 kg, while an AP placenta in human fetuses would be applied in smaller fetal sizes. This late gestational age range is adequate to assess the effects on lung development equivalent to 24 weeks in humans, but prevents evaluating the effects on other important aspects, including those related to size and cardiovascular adaptation. We have performed several experiments on 95-day lamb fetuses (around 600 g) with apparently similar success rates, but this group of cases was smaller, and for consistency in this study, we reported only fetuses of 110–115 days. The study used mainly components adapted from other medical uses, and we acknowledge that some of the mechanical problems here mentioned are most likely solved with purpose-designed material. Finally, we also acknowledge that due to the descriptive nature of this manuscript, the number of cases included in the subgroups evaluated did not allow robust statistical comparisons.

In conclusion, setting up and AP experimental prototype is extremely challenging, effort-consuming, and requires a multidisciplinary team. A true interdisciplinary combination of knowledge from fetal medicine, neonatology, intensive care, pediatric surgery, cardiology, and cardiovascular surgery is critical for success. In addition, a strong bioengineering team is required. The prototype here described can work well with components adapted from other clinical uses, but longer-term survival requires purpose-designed cannulae, oxygenator membranes, and EC circuits. An AP experimental system provides a unique research platform in preparation for future clinical applications and for other types of research in fetal development, physiology, and therapy.

## Figures and Tables

**Table 1:** Experimental outcomes according to survival goal group.

	1–3 h (n = 8)	4–24 h (n = 10)	48–168 h (n = 10)
Duration EXIT surgery (min)	33.5 ± 3.3	44.1 ± 5.1	34.3 ± 5.1
Heart rate before connection (bpm)	182 ± 18	168 ± 10	143 ± 6
Duration of cannulation (min)	8.04 ± 2.95	8.11 ± 0.93	6.89 ± 0.40
Cannulation technique used			
Direct cut-down	6 (75%)	0	0
Sequential technique	2 (25%)	5 (50%)	0
ST + vasospasm prevention	0	5 (50%)	10 (100%)
Complications surgical procedure	6 (75%)	5 (50%)	0
Bubbles in the circuit	2 (25%)	1 (14.3%)	-
Vasospasm	-	4 (57.1%)	-
Decannulation	3 (37.5%)	-	-
Vessel lesion	1 (12.5%)	-	-
Total survival time in AP system (hours)	1.48 ± 0.78	11 ± 3.42 *	72.6 ± 12.9 **
pH 30 min after connection	7.20 ± 0.06	7.31 ± 0.04	7.33 ± 0.02
Heart rate 30 min after connection (bpm)	134 ± 33	205 ± 8	182 ± 8
Circuit flow 30 min after connection (mL/kg/min)	45 ± 24	138 ± 23	196 ± 13 **
pH whole experiment	7.22 ± 0.07	7.29 ± 0.03	7.35 ± 0.01
Lactate whole experiment (mmol/L)	6.2 ± 1.22	3.82 ± 0.78	2.50 ± 0.38 **
Heart rate whole experiment (bpm)	222 ± 11	186 ± 9	177 ± 5
Circuit flow whole experiment (mL/kg/min)	124 ± 12	115 ± 11	161 ± 8
Complications after 24 h survival ^#^	N/A	N/A	4 (40%)

Values are means ± SEM or n (%) * *p* < 0.05 1 h vs. 3–24 h; ** *p* < 0.05 1 h vs. 48–168 h. ^#^ Only in fetuses with >24 h survival.

## Data Availability

Not applicable.
